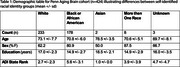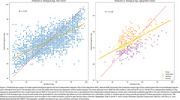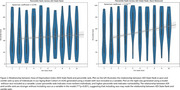# Exploring the Influence of SSDOH and Self‐Identified Race on Resilience and Vulnerability using Machine Learning for NACC UDS

**DOI:** 10.1002/alz70857_105739

**Published:** 2025-12-25

**Authors:** Emma P Fischer, Zahra Khodakarami, Christopher A Brown, Dawn Mechanic‐Hamilton, Paul A. Yushkevich, Sandhitsu R. Das, David A. Wolk

**Affiliations:** ^1^ University of Pennsylvania, Philadelphia, PA, USA

## Abstract

**Background:**

Current cognitive analysis methods in neurodegenerative disease rely on the use of individual neuropsychological assessments, which can be subject to variation and bias. A potentially more robust way to represent cognitive status is to use a cognitive age model to predict an individual's age from their cognitive assessment scores and demographics. This modeling allows for identification of individuals exhibiting resilience or vulnerability to normal aging processes across diverse datasets.

**Method:**

Psychometric and demographic data from cognitively unimpaired individuals from NACC UDS 3 (*n* = 11,752) were obtained. Neuropsychological assessment and demographic data were used to build a random forest model to predict a “cognitive” age. The distributions of predicted cognitive ages for each biological age were used to calculate a percentile rank for individuals in an independent dataset, with higher and lower percentile rank indicating vulnerability (cognitive > chronologic age) and resilience (cognitive < chronologic age), respectively. We assessed this model both with and without self‐identified race (white, black) as a predictor variable and related percentile rank to Area of Deprivation Index (ADI).

**Result:**

We applied the model to cognitively unimpaired individuals in the Penn Aging Brain Cohort (*n* = 424). The model without race as a predictive feature resulted in a stronger relationship between “cognitive” age centile rank and ADI, suggesting that covarying for race may partially mask the effects of ADI. Removing race from the model also resulted in higher percentile ranks for Black participants, indicating a higher level of vulnerability in these individuals. Percentile rank can also be used to place individuals into resilient, normal, and vulnerable groups to understand what factors contribute to resilience and vulnerability. Preliminary analyses of structural MRI data comparing resilient and vulnerable groups yielded moderately higher thickness in the anterior cingulate cortex and medial frontal cortex of resilient individuals, necessitating further research in a larger population.

**Conclusion:**

This machine learning model is a valuable tool for analyzing cognition and identifying abnormal aging patterns in large‐scale datasets, including identification of vulnerable and resilient individuals. Understanding the influence of demographic variables in generating these predictions allows us to better understand their role in vulnerable and resilient aging.